# Bronchobiliary fistula after traumatic liver rupture: a case report

**DOI:** 10.1186/s13256-024-04620-1

**Published:** 2024-06-24

**Authors:** Teng Zhou, Wenming Wu, Chao Cheng, Hui Wang, Xiaochuan Hu, Zhenhui Jiang

**Affiliations:** Department of Hepatobiliary Surgery, Huangshan City People’s Hospital Affiliated to Wannan Medical College, Huangshan, China

**Keywords:** Bronchobiliary fistula, Trauma, Liver rupture

## Abstract

**Introduction:**

Bronchobiliary fistulas are rare and difficult to treat. Peacock first reported this entity in 1850 while treating a patient with hepatic encopresis.

**Case presentation:**

A 67-year-old Chinese male patient presented to the outpatient clinic with a complaint of coughing up phlegm with chest tightness for 4 days with symptoms of intermittent bilirubin sputum with a sputum volume of about 500 ml per day but no symptoms of abdominal pain or jaundice and no yellow urine or steatorrhea. The examination revealed cyanosis of the lips and mouth, barrel chest, low breath sounds on the right side, and a large number of wet rales heard in both lungs. The imaging investigations were suggestive of bronchobiliary fistula. Therefore, the patient was operated on and discharged with no perioperative complications.

**Conclusion:**

Bronchobiliary fistula should be considered diagnostically in patients with known liver disease who also experience trauma or medical treatment and cough up bile-colored sputum, regardless of the presence of concurrent infections, and in conjunction with radiological expertise to identify it. Here, we report a case of bronchobiliary fistula and a brief review of the literature on it.

## Introduction

Bronchobiliary fistula (BBF) is a fistula deformity due to the formation of a pathological connection between the bronchus and the bile duct. Coughing up bile is a characteristic feature of BBF. Intermittent bile-colored sputum is its specific sign and has diagnostic significance [1]. The volume of sputum ranges from 50 to 1200 ml per day. The time of onset of bile sputum is related to the etiology of fistula formation.

BBF can be divided into congenital and acquired according to the cause of its formation. The former is common in childhood, especially in infants and young children [2], and the site of onset is often the same as that of adults, with the right lung being the most common and often solitary. The pathogenesis of congenital BBF is unclear and may be related to the following factors: (1) the presence of specific malformations in the upper gastrointestinal tract, with an abnormal connection between the trachea and the intrahepatic bile ducts; and (2) an abnormal connection between the lung tissues and the liver tissues [3]. It is more common in females and is characterized by respiratory symptoms and recurrent lung infections. The causes of acquired BBF are numerous and complex. The common causes of acquired BBF are biliary obstruction and infection. Stones and strictures are the causes of biliary obstruction, especially acute obstructive septic cholangitis that occurs with hepatic duct stones, roundworms, and strictures.

The disease is rare clinically and highly susceptible to underdiagnosis and misdiagnosis, which can lead to poor prognosis for the patient [4]. Understanding this disease is necessary for surgical planning, the appropriate interpretation of intraoperative surgical findings, and the design of postoperative therapy.

## Case report

A 67-year-old Chinese male patient presented to the outpatient clinic with a complaint of coughing up phlegm with chest tightness for 4 days with symptoms of intermittent bilirubin sputum with a sputum volume of about 500 ml per day but no symptoms of abdominal pain or jaundice and no yellow urine or steatorrhea. The patient was treated for traumatic hepatic rupture with hepatic repair surgery 3 years previously, and the trauma site was located in segments IV, V, and VIII of the liver. The patient had no history of smoking or any risk factor suggesting lung abscess. He had no history of diabetes, hypertension, asthma, or any known drug allergy.

The patient's temperature and blood pressure were in the normal range on examination. His body mass index (BMI) was 22 kg/m^2^. At presentation, he was in poor spirits, with cyanosis of the lips and mouth, barrel chest, low breath sounds on the right side, and a large number of wet rales heard in both lungs. The heart rate was 123 beats per minute and rhythmic, and no apparent murmurs were heard. The rest of the examinations were unremarkable. The laboratory results showed respiratory alkalosis with low partial pressure of oxygen, high inflammatory indicators, and hypokalemia (Table 1).

**Table 1:** Laboratory tests at admission

Laboratory measures	Reported values	Normal range
White blood cell count	22.95 × 10^9^	4.00–10.00 × 10^9^/L
Neutrophil count	21.90 × 10^9^	2.00–7.00 × 10^9^/L
Procalcitonin	8.81	0.00–0.25 ng/ml
C-reactive protein	219.80	0.00–10.00 mg/L
pH	7.503	7.35–7.45
PO_2_	61.9	80–100 mmHg
PCO_2_	32.5	35–45 mmHg
Lac	2.8	0.5–1.7 mmol/L

*pH* potential hydrogen, *PO*_*2*_ partial pressure of oxygen, *PCO*_*2*_ partial pressure of carbon dioxide, *Lac* lactic acid

**Fig. 1:**
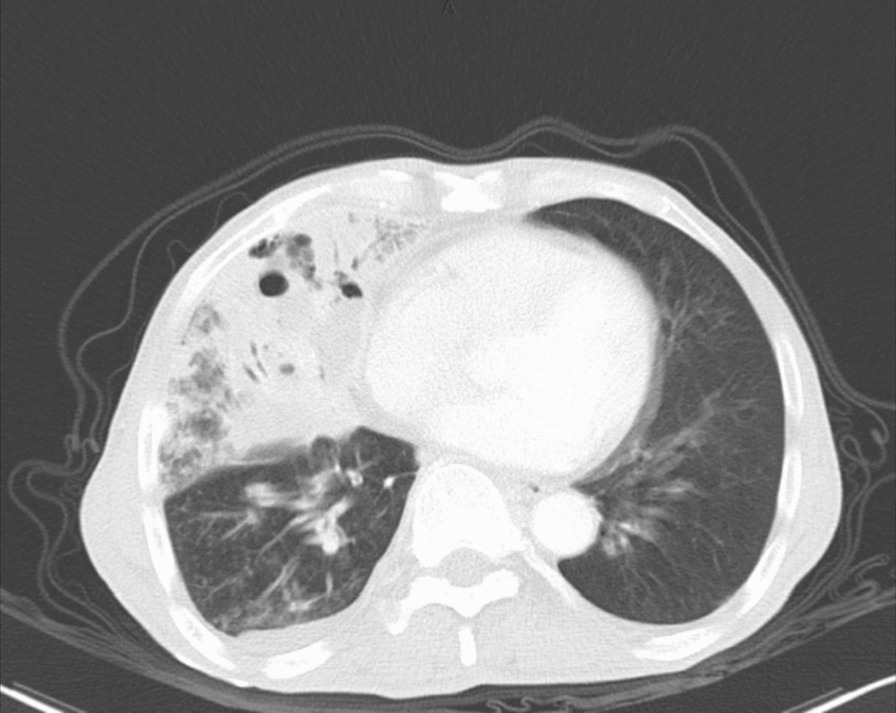
Chest CT scan showing sizable solid shadow in the middle and lower lobes of the right lung, large right pleural effusion with incomplete expansion of the right lower lung under pressure

**Fig. 2:**
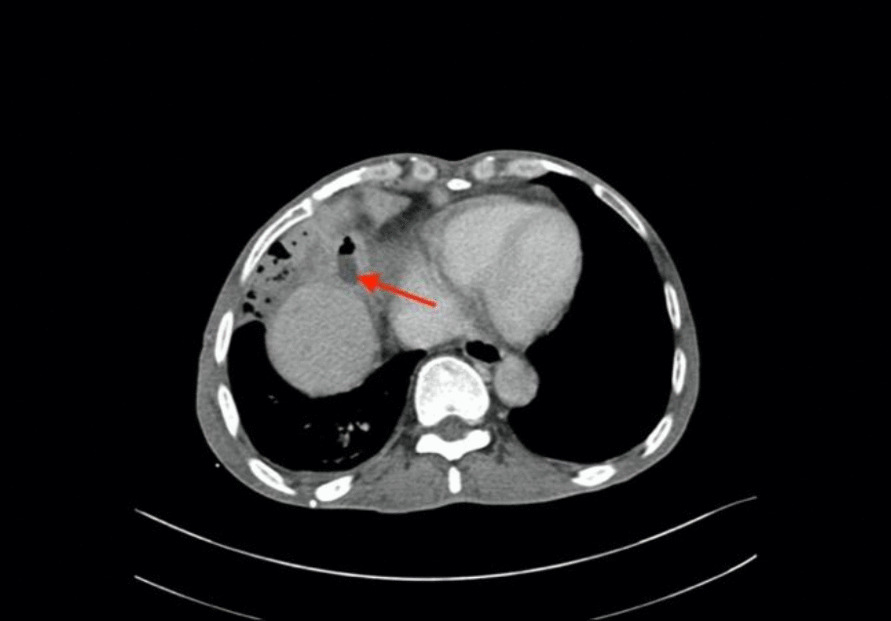
The BBF seen on the CT scan of the abdomen (red arrow)

**Fig. 3:**
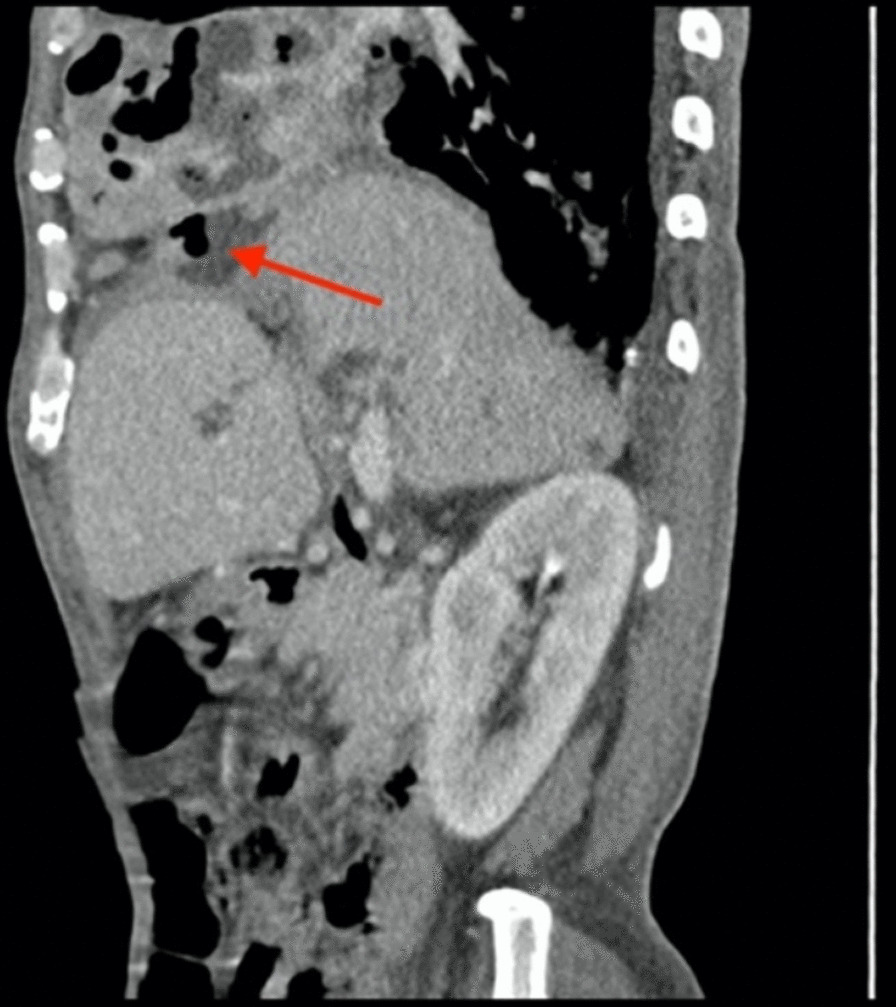
The BBF on the sagittal CT scan of the abdomen (red arrow)

**Fig. 4:**
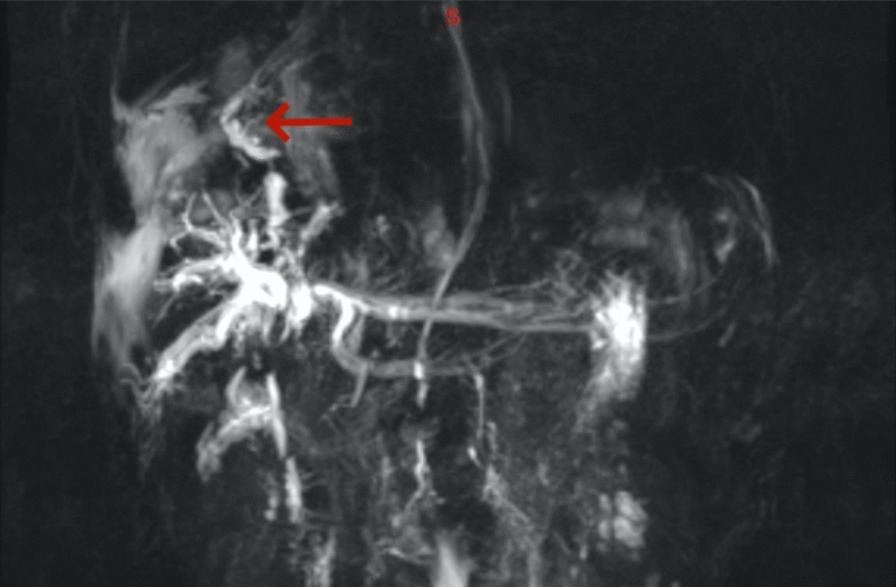
The BBF on MRCP (red arrow)

Computed tomography (CT) revealed a large solid shadow in the middle and lower lobes of the right lung and a large right pleural effusion with incomplete expansion of the right lower lung under pressure. Multiple lymph nodes in the mediastinum, enlarged right subdiaphragmatic lymph nodes, cirrhotic manifestations, slightly hypodense areas, and small foci of translucent shadows were seen in the subperiosteum and intrahepatic area, infectious lesions, and slight dilatation of the intrahepatic bile ducts BBF (Figs. 1, 2, 3). Magnetic resonance cholangiopancreatography (MRCP) displayed the path of the bile ducts, especially the BBF (Fig. 4).

The patient was admitted to the respiratory medicine department, where he was treated with anti-infection (cefoperazone sulbactam) and right-sided chest drainage to improve proper lung compression. After a multidisciplinary consultation (respiratory medicine, radiology, and surgeon), the patient then underwent an explorative laparotomy with a right upper quadrant subcostal incision. The patient was operated on under general anesthesia by a hepatobiliary surgeon and general surgery residents. The findings are as detailed here. We observed that the entire upper abdominal cavity was densely adherent, with the transverse colon, duodenum, and gastric sinusoids adhering to the liver, dense adhesions between the visceral surface of the liver and the diaphragm, post-cholecystectomy changes, and atrophy of segments IV, V, and VIII of the liver, with dense adhesions in the porta hepatis, which made it impossible to separate them, and hypertrophy of the left hepatic outer lobe, with a relatively atrophied right posterior lobe, which was considered to be caused by the patient’s posttraumatic atrophy of the liver. The right liver was adequately freed, and the adhesions between the liver and the diaphragm were separated first; after separation, a sinusoidal tract was seen, and the diaphragm in its vicinity was stiff, and the surrounding tissues were markedly edematous, and bile was seen to overflow from the surface of the atrophied liver after separation, which was considered to be the location of the bile duct leakage. We closed the rupture of the liver with 4–0 provolone thread to close the broken bile duct, then adequately repaired the rupture of the right diaphragm and placed drainage in the abdominal cavity. The patient’s bile culture showed *Pseudomonas aeruginosa*, which was multidrug resistant. We placed the patient in an isolation ward, and all gauze changes were handled separately, and discharging the patient without perioperative complications was carried out.

**Fig.5:**
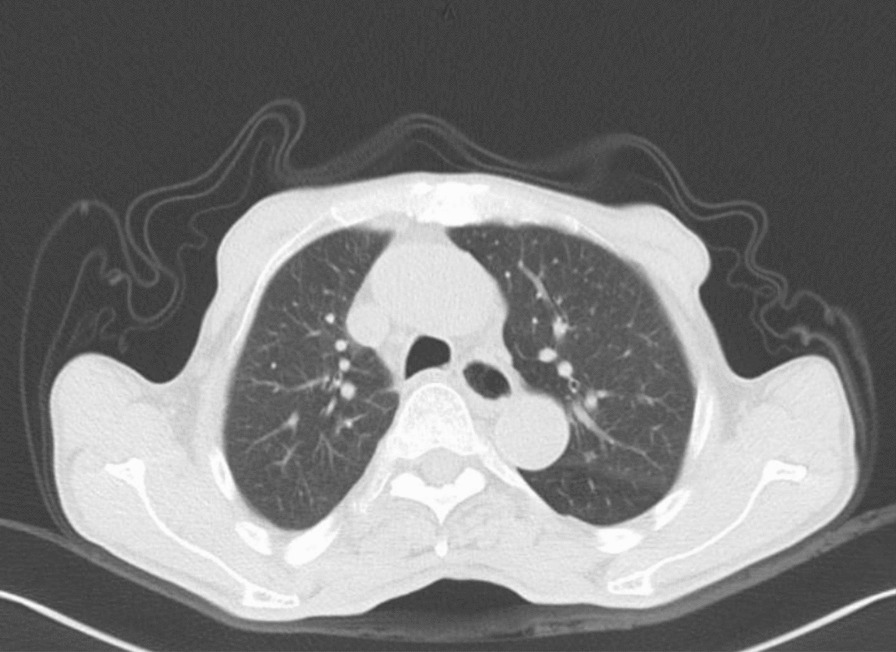
Chest CT at patient follow-up

The patient’s postoperative course was smooth. He was discharged on the third day after all the tubes were removed. He had come to our outpatient department every 3 months for checkups and to monitor his liver function and inflammatory markers. A follow-up period of 1.5 years was completed (Fig. 5).

## Discussion

BBF can cause coughing up of bile-colored sputum, even though the standard abdominal and thoracic cavities are not connected, and bile does not usually flow from the trachea. Peacock [5] first reported BBF in 1850 while treating a patient with hepatic encopresis. It is a rare but severe complication of diseases of the hepatobiliary system, with a high morbidity and mortality rate.

However, the typical symptom of BBF is intermittent sputum that resembles bile. Early diagnosis can be challenging, as it may be mistaken for yellow pus sputum caused by aspiration pneumonia resulting from esophageal reflux. Intermittent bilirubin sputum is a specific manifestation of this disease and has diagnostic significance. A large percentage of patients present with clinical signs of pneumonia, such as a frequent, irritating cough, with or without fever, jaundice, abdominal pain, shortness of breath, and dyspnea. The amount of sputum varies from tens to thousands of milliliters per day. The timing of the onset of bilious sputum correlates with the etiology of fistula formation. The typical color of sputum is bright yellow or yellow−green. A positive laboratory test for bilirubin can be used to diagnose the condition [6]. The bacteria commonly found in patients’ sputum tests are consistent with the spectrum of common biliary bacteria, including *Escherichia coli*, *Pseudomonas aeruginosa*, *Klebsiella pneumoniae*, *Enterococcus*, and *Enterobacter cloacae* [7]. Complications such as pneumonia, lung abscess, pleural effusion, and respiratory failure are common.

The primary diagnostic methods for BBF are imaging studies, such as CT scans and the examination of bilirubin sputum. CT scans reveal an accumulation of fluid under the diaphragm, known as cholangioma, and fluid in the corresponding side’s pleural cavity, diaphragmatic tears, and bile duct dilatation. However, direct visualization of the fistula is challenging. MRCP has the advantage of providing clear visualization of the primary and secondary bile ducts [8] and any dilated bile ducts. On the other hand, endoscopic retrograde cholangiography (ERCP) is an invasive procedure that typically shows the contrast agent entering the right lower and right lower hemithoracic cavity. Despite its invasiveness, it offers irreplaceable advantages over other imaging tests [9]. The presence of a fistula can be directly visualized after the injection of the contrast medium, allowing for simultaneous diagnosis and treatment. Owing to the bile surge and narrow lumen, direct observation of the fistula is challenging. Bronchoscopy and bronchography have limited roles. Recently, a new type of contrast agent for hepatobiliary imaging has been utilized to diagnose BBF. One such agent is ^99m^Tc-mebrofenin, which can identify the site of biliary stasis and evaluate the anatomy and function of the biliary system in a noninvasive and repeatable manner. This is advantageous for diagnosing BBF, formulating therapeutic regimens, and evaluating therapeutic efficacy [10]. Treating BBF involves eliminating the lesion, closing the fistula, relieving the obstruction, and clearing the drainage. By eliminating or reducing the direct irritation of bile to the lungs, the amount of bile entering the bronchus through the pathologic fistula can be reduced.

Conservative medical treatment can be selected for patients with BBF on the basis of their history and condition. Surgical intervention should not be considered immediately. Only a few nonsurgical interventions have favorable prognosis [11]. Surgical procedures may include drainage of subphrenic abscesses, resection of fistulas and diseased liver and lung tissue, and reestablishment of bile drainage to the duodenum. In contrast, interventional therapy is especially appropriate for patients who are older or have weaker postsurgical condition owing to its minimally invasive and less risky advantages. The main methods of interventional treatment for BBF are biliary drainage, stent implantation, and fistula embolization [7]. Nonsurgical treatments for BBF have gained attention in recent years. Owing to the clinical rarity of BBF and the complexity of its etiology and formation mechanism, individualized treatment and multidisciplinary collaboration are the basic principles of treatment. The choice of treatment method should be based on individual patient needs and determined through collaboration among healthcare professionals from different disciplines. In this case, the patient developed a biliary fistula after liver trauma. Unfortunately, the patient could not follow up promptly due to the family’s remote location in a mountainous area with limited transportation. This delay resulted in the development of a postbiliary fistula cholangioma, which ultimately led to the formation of a BBF. While hospitalized in respiratory medicine, the patient was referred to hepatobiliary surgery owing to a large amount of sputum and the determination that the fistula could not be treated through bronchoscopic intervention.

The literature on BBF is limited, with only a few reported cases. A summary of the available literature after 2010 is presented in Table 2.

**Table 2:** Reported cases of BBF

Authors	Year of publication	Patient clinical presentation	Investigations	Objective of the paper
Zhen Bing, *et al.* [3]	2010	A 2-year-old boy presented with symptoms of cough and refractory pneumonia. There was no typical yellow−greenish sputum in the period of pneumonia. There was also no cyanosis, respiratory distress, or jaundice	Abdominal CT revealed a gas shadow in the liver. Bronchography allowed definite diagnosis of congenital BBF with the help of the bronchoscope and endobronchial blocker	This report describes the application of a double endobronchial blocker during operation, which can not only effectively implement one-lung ventilation for children but also help to determine the course of BBF and prevent bile acid reflux to the lung
Guan-Qun Liao *et al.* [9]	2012	A 31-year-old man presented with symptoms of continuous coughing and abundant yellowish−green sputum for 2 months but without signs of dyspnea or abdominal pain	ERCP showed contrast materials in the bile duct and bilateral thoracic cavity, confirming bronchobiliary fistula	This report describes a patient with BBF in the left lung, a case managed successfully without open surgery. Individualized and multidisciplinary treatment should be emphasized
Jonathan M. Harnoss *et al. *[1]	2013	A 45-year-old white male with PNH presented with right upper quadrant abdominal pain for 2 weeks	Ultrasound and CT showed subdiaphragmatic fluid compression near the right hepatic lobe, a wedge-shaped solid area in the right lower lung, and bronchiectasis	This report describes a patient with BBF with lithoptysis after ERCP and liver biopsy, emphasizing that surgical approach should be considered in such cases
Min Je Kim et al*.* [6]	2017	A 53-year-old male presented with fever, cough, and brownish sputum that had persisted for 2 months	A urine dipstick test revealed that the sputum was positive for bilirubin. Flexible bronchoscopy showed bile-colored secretion in the right lower lobar bronchus. A subprogram revealed a dilated bile duct and fistulous communication with the right bronchial tree	This report describes a patient with BBF developed after TACE for hepatocellular carcinoma, emphasizing that embolization with glue and Lipiodol of the fistula is an effective treatment option
Takeshi Matsumoto *et al.* [4]	2017	A 56-year-old woman presented with symptoms of repetitive cholangitis	Computed tomography showed consolidation with an air bronchogram in the right lower lobe	This report describes a patient with BBF who underwent living liver transplantation, emphasizing that a bronchoscopic biopsy of pneumonic lesions may be diagnostic even when the sputum findings are not bilious

*TACE* transarterial chemoembolization, *PNH* paroxysmal nocturnal hemoglobinuria

## Conclusion

Though rare, when encountered in the clinical setting, BBF requires thorough knowledge and advanced planning of treatment options. BBF should be considered diagnostically in patients with known liver disease who also experience trauma or medical treatment and cough up bile-colored sputum, regardless of the presence of concurrent infections, and in conjunction with radiological expertise to identify it. Increased awareness of this disease is the key to minimizing underdiagnosis. Our patient was diagnosed with acquired BBF due to liver trauma, and it has been reported as very rare.

## Data Availability

The datasets used and analyzed during the current study are available from the corresponding author upon reasonable request.
